# Reliability and validity of the Wolfram Unified Rating Scale (WURS)

**DOI:** 10.1186/1750-1172-7-89

**Published:** 2012-11-14

**Authors:** Chau Nguyen, Erin R Foster, Alexander R Paciorkowski, Amy Viehoever, Colleen Considine, Aidena Bondurant, Bess A Marshall, Tamara Hershey

**Affiliations:** 1 Program in Occupational Therapy, Washington University School of Medicine, St. Louis, Missouri, 63110, USA; 2 Department of Psychiatry, Washington University School of Medicine, St. Louis, Missouri 63110, USA; 3 Department of Neurology, Washington University School of Medicine, St. Louis, Missouri 63110, USA; 4 Department of Pediatrics, Washington University School of Medicine, St. Louis, Missouri, 63110, USA; 5 Department of Radiology, Washington University School of Medicine, St. Louis, Missouri 63110, USA; 6 Departments of Neurology, Pediatrics & Biomedical Genetics, Center for Neural Development & Disease, University of Rochester Medical Center, 601 Elmwood Avenue, Rochester, NY, 14642, USA

## Abstract

**Background:**

Wolfram syndrome (WFS) is a rare, neurodegenerative disease that typically presents with childhood onset insulin dependent diabetes mellitus, followed by optic atrophy, diabetes insipidus, deafness, and neurological and psychiatric dysfunction. There is no cure for the disease, but recent advances in research have improved understanding of the disease course. Measuring disease severity and progression with reliable and validated tools is a prerequisite for clinical trials of any new intervention for neurodegenerative conditions. To this end, we developed the Wolfram Unified Rating Scale (WURS) to measure the severity and individual variability of WFS symptoms. The aim of this study is to develop and test the reliability and validity of the Wolfram Unified Rating Scale (WURS).

**Methods:**

A rating scale of disease severity in WFS was developed by modifying a standardized assessment for another neurodegenerative condition (Batten disease). WFS experts scored the representativeness of WURS items for the disease. The WURS was administered to 13 individuals with WFS (6-25 years of age). Motor, balance, mood and quality of life were also evaluated with standard instruments. Inter-rater reliability, internal consistency reliability, concurrent, predictive and content validity of the WURS were calculated.

**Results:**

The WURS had high inter-rater reliability (ICCs>.93), moderate to high internal consistency reliability (Cronbach’s α = 0.78-0.91) and demonstrated good concurrent and predictive validity. There were significant correlations between the WURS Physical Assessment and motor and balance tests (r_s_>.67, p<.03), between the WURS Behavioral Scale and reports of mood and behavior (r_s_>.76, p<.04) and between WURS Total scores and quality of life (r_s_=-.86, p=.001). The WURS demonstrated acceptable content validity (Scale-Content Validity Index=0.83).

**Conclusions:**

These preliminary findings demonstrate that the WURS has acceptable reliability and validity and captures individual differences in disease severity in children and young adults with WFS.

## Background

Wolfram syndrome (WFS), also known as DIDMOAD (diabetes insipidus, diabetes mellitus, optic atrophy, and deafness), is an autosomal recessive neurodegenerative disease that typically presents with childhood onset insulin dependent diabetes mellitus. WFS disease is rare, with an incidence of 1 in 770,000 of the general population, and 1 in 150 of the population with insulin dependent diabetes mellitus [1,2]. WFS is associated with death by ages 30-50 [1-3], commonly due to respiratory failure. Other symptoms have been reported, including seizures, cognitive deficits, depression, bladder and bowel dysfunction and ataxia [3]. Although recent advances have improved the understanding of pathogenesis underlying the degenerative course in WFS [3], several aspects of the natural history of WFS are unclear. Measuring disease severity and progression with reliable and validated tools is a prerequisite for clinical trials of any new intervention for neurodegenerative conditions.

To this end, we developed the Wolfram Unified Rating Scale (WURS) to measure the severity and individual variability of WFS symptoms, with a particular focus on the known neurodegenerative aspects of the disease. This rating scale is based on established severity rating scales for other neurodegenerative conditions, such as Batten Disease, Parkinson disease and Huntington disease [4-6]. Such measures have proven to be critical for comparing samples across studies and centers, for understanding factors that influence disease severity and disease characteristics, and for the evaluation of interventions [4,6,7].

In order to establish the WURS as a potentially useful metric for disease severity and change in disease severity over time, we focus on the subscales that are meant to capture current symptom severity, with a particular emphasis on the neurological and neurodegenerative features of the disease. We describe the basic psychometric features of these scales, including inter-rater reliability, concurrent, predictive and content validity in a relatively small sample of WFS patients in the relatively early stages of the disease process. These preliminary findings support the reliability and validity of the current version of the WURS and indicate its potential for use in future larger and longitudinal studies.

## Methods

### Protocol

Testing of the WURS occurred at the Washington University School of Medicine Wolfram Syndrome Research Clinic at the Pediatric Clinical Research Unit (PCRU), St. Louis Children’s Hospital and Barnes-Jewish Hospital at Washington University in St. Louis. Patients with WFS were examined with the WURS, neurological and physical examinations, and psychiatric questionnaires to sample features thought to be affected by WFS. Blood glucose levels of the individuals with WFS were monitored and recorded by research personnel at various points throughout the Wolfram Syndrome Research Clinic. Informed consent from a parent for minor participants or from adult participants was obtained prior to participation in the research clinic. The study was approved by the Human Research Protection Office (HRPO) at Washington University in St. Louis.

### Participants

WFS patients aged 6 to 25 were recruited through the Washington University Wolfram Syndrome Registry website. Inclusion criteria were the diagnosis of insulin dependent diabetes mellitus and optic atrophy before the age of 18 and/or genetic confirmation of a *WFS1* mutation. Exclusion criteria were the patient being unaware of the diagnosis of WFS and advanced stage of the disease, making travel and participation difficult for the subject and/or family.

### WURS development

The WURS was developed in the following stages:

1. A review of the WFS literature was conducted to identify the most frequently reported symptoms of the disease.

2. Experts in WFS were identified to provide feedback on the WURS.

3. Initial psychometric properties were examined. The WURS was compared to a variety of physical and psychiatric assessments.

#### Stage 1

The WURS was modeled after the Unified Batten Disease Rating Scale (UBDRS) with the permission of the Neuronal Ceroid Study Group [4,7-10]. Batten disease is a neurodegenerative disease occurring in childhood that shares many symptoms with WFS, including vision loss, psychiatric abnormalities, and physical and neurological decline [4]. The UBDRS was developed to quantify symptom severity of Batten disease through ratings of physical, neurological, and psychiatric impairments, modeled on other standard clinical ratings scales for neurodegenerative disorders, such as the Unified Parkinson Disease Rating Scale (UPDRS) [6] and the Unified Huntington Disease Rating Scale (UHDRS) [5]. The UBDRS has been demonstrated to have good inter-rater reliability (ICCs = .68-.85) in studies of children with Batten disease [4]. In addition, the UBDRS Behavioral Assessment subscale has been cross validated with the Achenbach Child Behavior Checklist, with r_s_ ranging from 0.39 to 0.72 with related items [8]. The UBDRS has been used in studies to quantify decline in physical functioning [9], measure change in disease severity over time in longitudinal studies, to compare sex and genotype differences [8,10], and to determine treatment efficacy [11].

Given the acceptable metrics and utility of the UBDRS, and similar neurodegenerative features of Batten Disease and WFS, we retained all of the original items of the UBDRS for our first draft of the WURS. We then evaluated the literature for WFS specific symptoms that were not adequately covered by the UBDRS. On the basis of these reports, we added items on hearing, bladder function, bowel function, temperature regulation, tandem walking, and trunk stability [2]. Tandem walking was added to the WURS based on the standards of the Physical and Neurological Examination of Subtle Signs (PANESS) and Unified Huntington Disease Rating Scale (UHDRS) [12,13].

In this final form, the WURS was divided into the following subscales: Physical Assessment, Behavioral Assessment, Seizure Assessment, Capability Assessment, and WFS history.

The Seizure Assessment contains a descriptive questionnaire about the presence and frequency of various types of seizures experienced by the patient. Seizures are common in Batten’s disease, and since seizures have been reported in WFS, we elected to retain this section of the UBDRS in the WURS. The Capability Assessment contains 10 items in which parents rate their child’s ability to take part in school, chores, play, activities of daily living, and chores. The individual is rated on capability with these activities, including and excluding functional impairment resulting from visual deficits. The WFS History section allows the rater to document the age of onset of existing classical WFS symptoms and any additional symptoms that are not listed.

We will report here on the two main subscales that are meant to capture current severity of neurological symptoms in WFS and thus are most suited for tracking changes over time: Physical Assessment (34 items rated on a scale from 0 = no symptoms to 4 = highest severity; total score range = 0-136) and Behavioral Assessment (9 items rated on frequency and severity from 0 = normal behavior to 3 = highest severity; total score range = 0-54). The Physical Assessment is divided into two sections: One of which requires a physician to rate the physical symptoms observed in the patient, and the other in which the parent reports the severity of their child’s symptoms that cannot be physically examined to the physician. A total score is generated through the summation of the Physical and Behavioral Assessment scores (43 items, range = 0-190). Individual items on the WURS Physical and Behavioral assessments are listed in Table 1. The Seizure and Capability assessments will be examined in future studies.

**Table 1:** WURS Domains and Items for Physical and Behavioral Assessments

**WURS Domain**	**Items**	**Maximum Score**
Physical – Physician Rated	Speech Clarity, Abnormal Repetitive Speech Sounds, Tongue Protrusion, Visual Acuity, Hearing, Passive Motion of Arms, Legs, and Neck, Power of Arms and Legs, Hand Taps, Maximal Dystonia, Normal Spontaneous Movements, Gait, Trunk Stability, Retropulsion Pull Test, Heel Stomping, Motor Tics or Stereotypes, Myoclonus, Rest Tremor, Tremor with Maintained Posture or Action, Dysmetria, Appendicular Chorea, Tandem Walking	124
Physical – Parent Rated	Temperature Regulation, Bladder Control, and Bowel Control	12
Behavioral – Parent Rated	Sad Mood, Apathy, Anxiety, Aggression Toward Others, Aggression Toward Self, Stereotyped/Repetitive Behaviors, Compulsions, Auditory Hallucinations, and Obsessions	54
Total Score	Sum of Physical and Behavioral Assessments	190

The WURS in its entirety takes approximately 20 minutes to complete. For this preliminary study, only pediatric neurologists administered the exam. A training manual modeled after the UBDRS training manual was used to train the examiners.

#### Stage 2

Three content experts were chosen for their direct clinical experience with WFS and additional expertise in pediatric endocrinology (n = 1) or pediatric neurology (n = 2). These experts provided qualitative feedback on the organization, wording and format of the WURS.

#### Stage 3

**Additional tests administered**: Standard tests of physical, psychological, and neurological function were administered for comparison with the WURS Behavioral and Physical Scales in order to determine concurrent and predictive validity.

##### Physical and Neurological Examination for Subtle Signs (PANESS)

The PANESS assesses the presence of subtle neurologic signs in children through motor assessments. The sum of the Gaits & Stations portion of this assessment was used for our analyses and compared to age and gender based norms. Higher scores indicate greater severity with this assessment [12]. A pediatric neurologist administered the assessment.

##### Mini-Balance Evaluation Systems Test (MiniBESTest)

The Mini BESTest is a clinician rated assessment of dynamic balance. Lower scores are indicative of impairments in balance. The total possible score on this evaluation is a 32, indicating no balance impairment [14]. The assessment was administered by a physical therapist.

##### Achenbach Child Behavior Checklist (CBCL)

The Achenbach Child Behavior Checklist is a parent reported measure of aggressive behavior, anxiety, depression, attention problems, defiant behavior, social problems, somatic complaints, thought problems, and withdrawn behavior for ages 6 to 18. The CBCL Total Problems T Score was used in analyses (higher T scores indicate greater severity; borderline range = 60-64, clinical range ≥ 65 for Total T scores) [15].

##### Spence Children’s Anxiety Scale (SCAS)

The Spence Children’s Anxiety Scale is a self reported measure of anxiety for ages 8 to 15. It contains questions categorized into obsessive-compulsive problems, separation anxiety, social phobia, panic, agoraphobia, fears about physical injury, and generalized anxiety symptoms. T scores were calculated based on existing norms; a higher score indicates greater severity [16,17].

##### Pediatric Quality of Life Inventory, Generic Scales - Parent Report Version 4.0

The PedsQL^TM^ is a parent reported survey for children and young adults with subscales: Physical, Emotional, Social, and School Functioning [18]. The PedsQL Total score (sum of Physical and Psychosocial items), Physical score (sum of items on Physical subscale), and Psychosocial (sum of the Emotional, Social, and School subscales) scores were calculated. Higher scores indicate higher quality of life.

### Reliability

To test inter-rater reliability of the WURS, each patient was assessed by two pediatric neurologists simultaneously. One neurologist directed the patients to perform elements of the test and asked the parents questions for both the Physical and Behavioral Assessments, and then both neurologists scored the participants independently.

Inter-rater reliability of the WURS was determined through calculation of intraclass correlation coefficients for the Physical and Behavioral Assessments using mixed models and absolute agreement. Internal consistency reliability was determined through examination of Chronbach’s α.

### Content validity

Experts were asked to evaluate the WURS with a content validity form designed to calculate a Content Validity Index (CVI). Published guidelines were utilized to create the content validity form [19-21]. Individual items were rated on a scale of 1 to 4 to measure the perceived ‘representativeness’ of the Physical and Behavioral Assessment items to WFS. A score of 1 indicated that the item was judged to be not representative of WFS, 2 indicated that major revisions are recommended for the item, 3 indicated that minor revisions are recommended for the item, and 4 indicated that the item is representative of WFS and does not need revision.

The proportion of experts rating each item as relevant (score of 3 or 4 on the content validity rating form) was calculated for the Physical and Behavioral Assessments. These values were then averaged to compute the Scale Content Validity Index (S-CVI). Higher S-CVI values indicate greater content validity.

### Concurrent validity

The degree of concurrent validity between the WURS and other physical and psychological variables was determined using Spearman’s correlations on the WURS Total Score, WURS Physical Score, WURS Behavioral Score, PANESS, Mini-BESTest, CBCL, and SCAS. Spearman’s correlations were used to determine validity due to the ordinal rating scales utilized in all measures.

For each patient we calculated T scores on the CBCL and SCAS, z scores on the PANESS using published norms [12] and total scores on the Mini-BESTest. Spearman’s correlations were performed to determine how these measures related to appropriate WURS subscales.

### Predictive validity

In addition, total and domain scores were calculated for the PedsQL. Spearman’s correlations were calculated between the WURS Total Scale and the PedsQL Total, Physical, and Psychosocial scores to measure predictive validity of the WURS for health related outcomes.

## Results

### Reliability

Eleven participants were assessed by both raters with the WURS. Intraclass correlation coefficients (ICCs) were excellent for all scales of the WURS (Physical Rated ICC = 0.96, Physical Reported ICC = 0.93, Physical Total ICC = 0.97, Behavioral ICC = 0.97, and Total ICC = 0.98). The Physical and Behavioral Assessments on the WURS both had moderate to high internal consistency reliability (Cronbach’s alpha coefficient; Physical = 0.91; Behavioral = 0.78).

### Content validity

The Scale Content Validity Index (S-CVI) was 0.83 for the WURS Physical and Behavioral Assessments combined. The minimum acceptable value for the S-CVI of a measure is 0.80 [19].

### Concurrent and predictive validity

**Figure 1:**
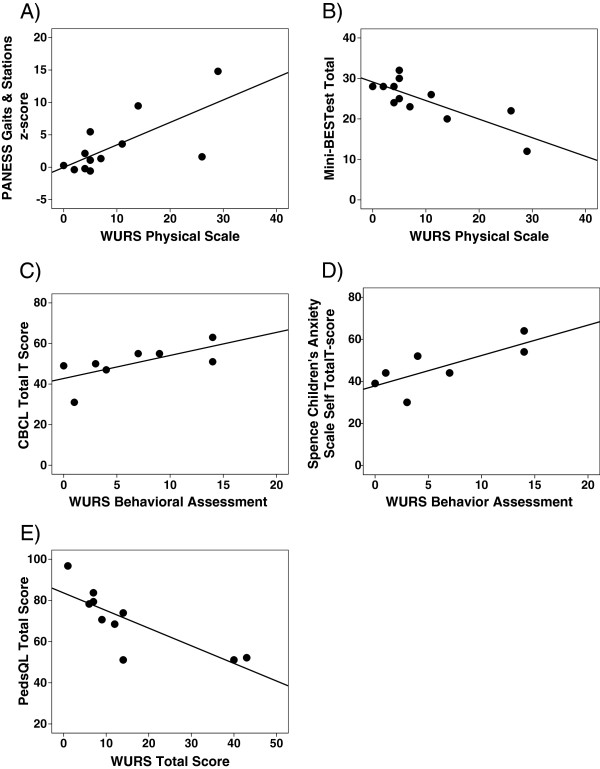
**Scatterplots of WURS scales vs. other measures: A) PANESS Gaits & Stations z-score and WURS Physical Scale (r**_**s**_**= 0.67, p = 0.02) B) Mini-BESTest Total scores & WURS Physical Assessment (r**_**s**_**= -0.69, p = 0.01) C) CBCL Total T score and WURS Behavioral Assessment (r**_**s**_**= 0.77, p = 0.03) D) SCAS Total T score and WURS Behavioral Assessment (r**_**s**_**= 0.80, p = 0.03) E) WURS Total & PedsQL Parent Total Scores (r**_**s**_**= -0.86, p = 0.001).**

Twelve participants were able to complete both the WURS and motor and balance tests. WURS results are reported in Table 2. Higher (more severely affected) scores on the WURS Physical scale (but not the Behavioral scale) correlated with poorer scores on the PANESS and Mini-BESTest, but not the CBCL and SCAS (Table 3 and Figure 1A-D).

**Table 2:** Median, Standard Deviation, and Range of scores on the WURS

**WURS Domain**	**Median**	**Min**	**Max**
Physical Assessment	5	0	29
Behavioral Assessment	3.5	0	14
Total Score	11.5	3	40

**Table 3:** Intercorrelations among WURS and other measures of severity

	**Mini-BESTest (n=12)**		**PANESS (n=12)**		**CBCL (n=8)**		**SCAS (n=8)**		**PedsQL Total (n=10)**	
	**r**_**s**_	**p**	**r**_**s**_	**p**	**r**_**s**_	**p**	**r**_**s**_	**p**	**r**_**s**_	**p**
WURS Physical	−0.69	0.01	0.67	0.02	0.31	0.46	0.41	0.36		
WURS Behavioral	−0.12	0.71	−0.10	0.77	0.77	0.03	0.80	0.03		
WURS Total	−0.54	0.07	0.45	0.14	0.62	0.10	0.69	0.09	−0.86	0.001

Z-scores on the PANESS ranged from -0.38 to 14.8, with higher scores indicating greater impairment, while raw scores on the Mini BESTest ranged from 12 to 32, with lower scores indicating higher severity of symptoms. Higher (more severely affected) scores on the WURS Behavioral Assessment (but not the Physical Scale) correlated with higher (more severely affected) scores on the CBCL and SCAS scales (Table 3 and Figures 1A-B). For the CBCL, 2 of the 8 subjects scored within the clinical or borderline range of severity. For the SCAS, 1 of the 8 subjects demonstrated elevated scores of anxiety. Higher scores on the WURS Total Scale, indicating more severity of symptoms, also correlated with lower scores (more severely affected) on the PedsQL (Table 3, Figure 1E).

Mean blood glucose levels taken during the study for the individuals with WFS averaged 222.5 mg/dL (SD = 69.1 mg/dL, max = 312.5 mg/dL, min = 94 mg/dL). No patients experienced any acute neurological symptoms related to hypoglycemia or hyperglycemia during the study.

## Discussion

The findings of this study confirm the reliability and validity of this version of the WURS and suggest that it may be useful as a tool for assessing severity across neurological, physical and behavioral domains in WFS. The WURS demonstrates good inter-rater reliability and internal consistency, supporting the potential utility of the WURS across both clinical and research settings, including clinical trials. Content and concurrent validity were acceptable, as indicated by the high S-CVI values and Spearman’s rho. This demonstrates that the current version of the WURS is an accurate measure of many of the key neurological aspects of WFS. In addition to concurrent validity, we also found that the WURS had good predictive validity, in that it correlated with overall quality of life. Quality of life is considered to be the most important indicator of health outcomes in clinical practice and research. It is also considered by the FDA to be the most relevant outcome measure for clinical trials [18,22,23]. Thus, these preliminary data suggest that the WURS is capturing aspects of the disease that are important to an individual’s daily life and well-being.

The selective nature of the correlations between WURS scales and conceptually similar standardized measures also suggested that the Physical and Behavioral scales capture qualitatively different constructs. For instance, the WURS Physical Assessment correlated highly with physical and neurological measures, but had low correlations with psychiatric measures. The WURS Behavioral Assessment demonstrated high correlations with measures of mood and behavior, and low correlations with physical and neurological assessments.

Furthermore, results suggest that there is variability within WFS for each construct that may be important to capture. For example, some aspects of WFS may progress at a different rate than others, or be differentially responsive to treatment. Thus, having valid, reliable measurement tools for these different constructs may be highly clinically relevant [20]. Longitudinal follow-up of this cohort and a larger, more diverse sample will be necessary to determine whether this is true.

The WURS appears to be sensitive to relatively mild stages of WFS. Our sample was small and tended to be in the early stages of the disease, but still had some variation in duration and severity of symptoms. The WURS captured this variability, distinguishing between mild and moderate levels of severity. For instance, the range of scores on the WURS Behavioral assessment (0 to 14 out of a possible 54) indicated that the individuals tested had relatively subtle behavioral issues. Yet, these scores correlated with ratings on the CBCL and SCAS, which placed the majority of participants within the normal range according to published norms. The mild levels of severity of all assessments of mood and behavior, in addition to the significant correlations between these measures, demonstrate that the WURS Behavioral Assessment is a sensitive measure of mood and behavior problems. The Mini-BESTest and PANESS, measures of physical and neurological impairment, also correlated highly with the WURS Physical Scale. Scores on these assessments demonstrated low to moderate levels of impairment indicating that this domain is also an accurate and sensitive measure of physical impairment.

There are several limitations of this study, including the small sample size, which is not uncommon in clinical research studies of rare disease. Continued research in this population across research centers, clinics and countries using standardized assessments such as the WURS will be necessary to draw stronger conclusions. Given the now promising validity and reliability of the WURS, we are now starting to establish such collaborations. In addition, time constraints limited us from having both WURS raters independently administer and score the WURS. To eliminate potential of bias by having both raters in the same room, future studies should implement independent administration and scoring of the WURS. Despite this limitation, our method does allow the conclusion that the current version of the WURS can be used consistently across individuals when they are provided with the same information.

Future modifications of the WURS are possible. For example, the WURS currently lacks some reported symptoms of WFS, including dysphagia, gag reflex impairment, peripheral and autonomic neuropathy, and cranial nerve involvement [24,25]. These items could be added to the WURS Physical Assessment, which may be particularly useful with more severely affected cohorts. Notably, there is a part of the WURS that allows for symptoms not listed to be noted by the rater. In our sample, none of these symptoms were noted. The WURS also includes items from the UBDRS that are not as widely recognized as common WFS symptoms. At this stage of the WURS development, we decided to keep all items from the UBDRS because they were psychometrically sound, and we could not be sure that the items would not be useful in more severely affected patients. With greater use of the WURS across time, a wider range of age and severity levels, we will be able to perform item specific analyses and drop items that have no added value for assessing disease severity and change over time. Finally, with the exception of the WFS History section, diabetes-related symptoms are not specifically addressed in the WURS. It is thought that these symptoms are not degenerative after diagnosis, unlike those of the neurological aspects of disease. However, exploration of diabetes-related symptoms may be necessary for a better understanding of diabetes treatment issues. Appropriateness of diabetes related items will be considered for the WURS once further examination is completed. Importantly, there are existing validated questionnaires for diabetes complications, management and quality of life that could be used as adjunct measures to the WURS if desired [26-29]. Similarly, cognitive testing is not currently incorporated into the WURS. While cognitive impairment has been reported in advanced WFS, it has not been found with standard assessments in our small sample of early WFS patients [30]. Further assessment will determine if cognitive items should be added to the WURS, or if the result of a supplementary cognitive test should be reflected in the WURS scoring.

Although neurologists are currently the primary clinicians using the assessment, it is hoped that other health care practitioners will be able to administer the WURS effectively. A training video will be developed in the future. We will investigate the minimum amount of training necessary for neurologists or other health professionals to administer the WURS. The UPDRS and other similar ratings scales are typically given by a variety of health professionals and technicians, who follow standardized training.

In summary, the preliminary data presented here support the use of the WURS in studies of WFS as a reliable and valid measurement tool for disease severity. The scale will continue to be developed as we are able to test a larger and more diverse sample. Further development and analyses of the WURS will be important for continued research of WFS in order to better understand symptoms and progression of the disease, in addition to its eventual use in clinical trials for therapeutic interventions.

## Appendix

P. Austin, M.D. (Surgery), G. Earhart, Ph.D. (Physical Therapy), J. Hoekel, M.D. (Ophthalmology), T. Hullar, M.D. (Otolaryngology), R. Karzon, Ph.D. (Audiology & Communication Sciences), J. Lapp, B.A. (Pediatrics), J. Leey M.D. (Medicine), L. Manwaring, M.S. (Pediatrics), M.A. Permutt, M.D. (Medicine), K. Pickett, Ph.D. (Physical Therapy), J. Wasson B.S. (Internal Medicine), and N. H. White M.D., CDE (Pediatrics).

## Competing interest

The authors have no conflicts of interest to report.

## Authors’ contributions

CN contributed to the research design, data analysis and interpretation, and drafting of paper. ERF contributed to data analysis & interpretation, and critical review of the paper. ARP, AV, CC, AB, and BAM participated in the acquisition of data and critical review of the paper. TH contributed to the research design, data analysis & interpretation, drafting and critical review of paper, and approval of the submitted paper. All authors have read and approved the final manuscript.

## References

[B1] Barrett TG Bundey SE Macleod AF Neurodegeneration and diabetes: UK nationwide study of Wolfram (DIDMOAD) syndrome Lancet 1995 346 8988 1458 1463 10.1016/S0140-6736(95)92473-6 7490992

[B2] Ganie MA Bhat D Current developments in wolfram syndrome J Pediatr Endocrinol Metab 2009 22 1 3 10 19344068 10.1515/jpem.2009.22.1.3

[B3] Kumar S Wolfram syndrome: important implications for pediatricians and pediatric endocrinologists Pediatr Diabetes 2010 11 1 28 37 10.1111/j.1399-5448.2009.00518.x 20015125

[B4] Marshall FJ de Blieck EA Mink JW Dure L Adams H Messing S A clinical rating scale for Batten disease Neurology 2005 65 2 275 10.1212/01.wnl.0000169019.41332.8a 16043799

[B5] Kremer H Unified Huntington's disease rating scale: reliability and consistency Mov Disord 1996 11 2 136 142 8684382 10.1002/mds.870110204

[B6] Martinez-Martin P Gil-Nagel A Gracia LM Gómez JB Martínez-Sarriés J Bermejo F Unified Parkinson's disease rating scale characteristics and structure Mov Disord 1994 9 1 76 83 10.1002/mds.870090112 8139608

[B7] Cialone J Augustine E Newhouse N Vierhile A Marshall F Mink J Quantitative telemedicine ratings in Batten disease Neurol 2011 77 20 1808 1811 10.1212/WNL.0b013e3182377e29 PMC3233206 22013181

[B8] Adams HR Beck CA Levy E Jordan R Kwon JM Marshall FJ Genotype does not predict severity of behavioural phenotype in juvenile neuronal ceroid lipofuscinosis (Batten disease) Dev Med Child Neurol 2010 52 7 637 643 10.1111/j.1469-8749.2010.03628.x 20187884 PMC2895016

[B9] Kwon J Adams H Rothberg P Augustine E Marshall F Vierhile A Quantifying physical decline in juvenile neuronal ceroid lipofuscinosis (Batten disease) Neurol 2011 77 20 1801 1807 10.1212/WNL.0b013e318237f649 PMC3233207 22013180

[B10] Cialone J Adams H Augustine EF Marshall FJ Kwon JM Newhouse N Females experience a more severe disease course in batten disease J Inherit Metab Dis 2012 35 549 555 10.1007/s10545-011-9421-6 22167274 PMC3320704

[B11] Cialone J Augustine EF Newhouse N Adams H Vierhile A Marshall FJ Parent-reported benefits of flupirtine in juvenile neuronal ceroid lipofuscinosis (Batten disease; CLN3) are not supported by quantitative data J Inherit Metab Dis 2011 34 5 1075 1081 10.1007/s10545-011-9346-0 21556831 PMC3174318

[B12] Gidley Larson JC Mostofsky SH Goldberg MC Cutting LE Denckla MB Mahone EM Effects of Gender and Age on Motor Exam in Typically Developing Children Dev Neuropsychol 2007 32 1 543 562 2007/07/06 10.1080/87565640701361013 17650993 PMC2099302

[B13] Denckla MB Revised Neurological Examination for Subtle Signs (1985) Psychopharmacol Bull 1985 21 4 773 4089106

[B14] Franchignoni F Horak F Godi M Nardone A Giordano A Using psychometric techniques to improve the Balance Evaluation System’s Test: the mini-BESTest J Rehabil Med Offic J UEMS Eur Board Phys Rehabil Med 2010 42 4 323 10.2340/16501977-0537 PMC3228839 20461334

[B15] Achenbach TM Ruffle TM The Child Behavior Checklist and related forms for assessing behavioral/emotional problems and competencies Pediatr Rev 2000 21 8 265 10.1542/pir.21-8-265 10922023

[B16] Spence SH A measure of anxiety symptoms among children Behav Ther 1998 36 5 545 566 10.1016/S0005-7967(98)00034-5 9648330

[B17] Spence SH Barrett PM Turner CM Psychometric properties of the Spence Children’s Anxiety Scale with young adolescents J Anxiety Disord 2003 17 6 605 625 10.1016/S0887-6185(02)00236-0 14624814

[B18] Varni JW Burwinkle TM Seid M Skarr D The PedsQL 4.0 as a pediatric population health measure: feasibility, reliability, and validity Ambul Pediatr 2003 3 6 329 341 10.1367/1539-4409(2003)003<0329:TPAAPP>2.0.CO;2 14616041

[B19] Polit DF Beck CT The content validity index: Are you sure you know what's being reported? Critique and recommendations Res Nurs Health 2006 29 5 489 497 10.1002/nur.20147 16977646

[B20] Polit DF Beck CT Owen SV Is the CVI an acceptable indicator of content validity? Appraisal and recommendations Res Nurs Health 2007 30 4 459 467 10.1002/nur.20199 17654487

[B21] Grant JS Davis LL Selection and use of content experts for instrument development Res Nurs Health 1997 20 3 269 274 10.1002/(SICI)1098-240X(199706)20:3<269::AID-NUR9>3.0.CO;2-G 9179180

[B22] Varni JW Burwinkle TM Lane MM Health-related quality of life measurement in pediatric clinical practice: an appraisal and precept for future research and application Health Qual Life Outcome 2005 3 34 10.1186/1477-7525-3-34 PMC1156928 15904527

[B23] FDA Guidance for industry: qualification process for drug development tools: draft guidance 2010 Silver Spring, MD: Center for Drug Evaluation and Research

[B24] Mathis S Maisonobe T Neau JP Neuropathy in Wolfram syndrome Eur J Med Genet 2010 54 1 73 75 Jan-Feb 20888932 10.1016/j.ejmg.2010.09.011

[B25] Chaussenot A Bannwarth S Rouzier C Vialettes B Mkadem SAE Chabrol B Neurologic features and genotype-phenotype correlation in Wolfram syndrome Ann Neurol 2011 69 3 501 508 10.1002/ana.22160 21446023

[B26] Varni JW Burwinkle TM Jacobs JR Gottschalk M Kaufman F Jones KL The PedsQL™ in type 1 and type 2 diabetes Diabetes Care 2003 26 3 631 637 10.2337/diacare.26.3.631 12610013

[B27] Varni JW Burwinkle TM Seid M Skarr D The PedsQL™* 4.0 as a Pediatric Population Health Measure: Feasibility, Reliability, and Validity Ambul Pediatr 2003 3 6 329 341 10.1367/1539-4409(2003)003<0329:TPAAPP>2.0.CO;2 14616041

[B28] Fitzgerald JT Davis WK Connell CM Hess GE Funnell MM Hiss RG Development and validation of the Diabetes Care Profile Eval Health Prof 1996 19 2 208 230 10.1177/016327879601900205 10186911

[B29] Boyer JG Earp JAL The development of an instrument for assessing the quality of life of people with diabetes: Diabetes-39 Med Care 1997 35 5 440 10.1097/00005650-199705000-00003 9140334

[B30] Hershey T Lugar HM Shimony JS Rutlin J Koller JM Perantie DC Early Brain Vulnerability in Wolfram Syndrome PLoS One 2012 7 7 e40604 10.1371/journal.pone.0040604 22792385 PMC3394712

